# Inferences of associated latent variables by the observable test scores

**DOI:** 10.1111/bmsp.70002

**Published:** 2025-06-18

**Authors:** Rudy Ligtvoet

**Affiliations:** ^1^ University Hospital Cologne Cologne Germany

**Keywords:** monotone likelihood ratio, sum score, variables being associated

## Abstract

Test scores, like the sum score, can be useful for making inferences about the latent variables. The conditions under which such test scores allow for inferences of the latent variables based on a “weaker” stochastic ordering are generalized to any monotone latent variable model for which the latent variables are associated. The generality of these conditions places the sum score, or indeed any test score, well beyond a mere intuitive measure or a relic from classical test theory.

## 1 INTRODUCTION

Latent variable models for multiple item scores are useful to the extent that they allow for inferences to be made about the latent variables $\Theta_{1},\text{…},\Theta_{d}$, that are assumed to describe the dependencies that exist between the item scores. Many parametric latent variable models are available that allow for such inferences. A downside of these models is their reliance on parametric assumptions that are usually made based on pragmatic considerations of mathematical convenience. Hereto, a notable exception is the derivation of the Rasch (1960) model from the principle of *specific objectivity* (Rasch, 1977), for which the parametric model characteristics follow from requiring the sum score across items to be a sufficient statistic for the *unidimensional* latent variable (i.e., d=1; Andersen, 1977; Fischer, 1974). Results of testing are in practice also communicated in terms of the same sum score, but often without the empirical validation of any particular latent variable model. Unlike the parametric model restrictions, this presents a pragmatic consideration of a different kind, namely the convenience with which the sum score on a test can be computed and communicated. Such use of the sum score is often referred to as measurement by *fiat* (Torgerson, 1958) and a relic from a classical treatment of test scores (e.g., McNeish & Wolf, 2020; Sijtsma et al., 2024; Widaman & Revelle, 2023, for a discussion). However, the assessment of the sum score as a mere intuitive measure ignores the bulk of evidence that has been accumulated in favour of the use of the sum score for making inferences in the context of latent variable models. For example, Ellis and Junker (1997) provided a fully observable characterization of a general class of latent variable models for an infinite sequence of random item variables $\left(X_{1},X_{2},\text{…}\right)$, whereby the latent variables can be consistently estimated from any sequence with an arbitrary number of random item variables omitted. This work is closely related to the concept of *essential unidimensionality* of the sequence $\left(X_{1},X_{2},\text{…}\right)$, where a dominant latent variable can be consistently estimated by the sum score (Junker, 1991; Stout, 1990). For a finite number of n random item variables $X_{1},\text{…},X_{n}$, the focus has been on attaining a *stochastic ordering* of the unidimensional latent variable Θ by the sum score $S=X_{1}+\text{⋯}+X_{n}$. This property is referred to as SOL (Hemker et al., 1997) and states that, for all θ,

P[Θ>θ|S=s]is non‐decreasing ins.

As SOL is hard to establish directly, a more restrictive property called a *monotone likelihood ratio* (MLR) is usually considered instead. The *congeneric one‐factor model* (Jöreskog, 1971) is an example of a model for normal item scores that implies this MLR (Ligtvoet, 2022a), and thus allows for a stochastic ordering on the latent variable Θ by the sum score S. Mokken's (1971) model of *monotone homogeneity* for binary item variables, and special cases thereof, like the *two‐parameter logistic model* (Birnbaum, 1968), the *normal ogive models* (Lord, 1952, 1980), and the Rasch (1960) model, all imply an MLR (Grayson, 1988; Ünlü, 2008). However, Hemker et al. (1996, 1997) found that most of the latent variable models that are used in practice for polytomous item scores do not imply an MLR, which include the *graded response model* (Samejima, 1969), the *generalized partial credit model* (Muraki, 1992), and the *sequential model* (Tutz, 1990). This suggests that the restrictions imposed by the MLR property may be too restrictive. To mitigate the restrictive nature of an MLR, Van der Ark and Bergsma (2010) proposed a “weaker” version of SOL, and showed that it is implied by all the above mentioned latent variable models.

In the following, two results are discussed that support the use of the test score for making stochastic inferences. The first result is by Ghurye and Wallace (1959), who provide sufficient conditions for an MLR. The result by Jogdeo (1978) provides the conditions under which any test score that is a non‐decreasing function of the item variables, including the sum score, implies a weak SOL for any non‐decreasing function of the latent variables $\Theta_{1},\text{…},\Theta_{d}$. This second result considerably generalizing the class of latent variable models that allow for a stochastic ordering by the test score. More specifically, Theorem 2 presents the general (non‐parametric) conditions under which the observable test score is associated with any and each of the multi‐dimensional latent variables, thus placing the sum score well beyond a mere intuitive measure or a relic from classical test theory.

## 2 STOCHASTIC ORDERINGS ON LATENT VARIABLES

The first result on the stochastic ordering of the unidimensional latent variable Θ by the sum score S, in accordance to an MLR, was obtained by Ghurye and Wallace (1959).

### 2.1 A monotone likelihood ratio

Let f be a real‐valued positive function defined on 34𝒳×ℝ, where 34𝒳 is an ordered set and f(u,θ) is taken to be measurable in u for each θ. We say that the function f is (latent) *totally positive of order 2* (TP; Holland & Rosenbaum, 1986; Karlin, 1968), if for all $u_{1},u_{2}\in \mathrm{34𝒳}$ with $u_{1}\leq u_{2}$, and all $\theta_{1}\leq \theta_{2}$,

$$f(u_{1},\theta_{1})f(u_{2},\theta_{2})\geq f(u_{1},\theta_{2})f(u_{2},\theta_{1}).$$

Further, we say that f is a *Pólya frequency function of order 2* (PF; e.g., Efron, 1965), if for all $u_{1},u_{2},u_{3},u_{4}\in \mathrm{34𝒳}$ with $u_{1}\leq u_{2}$ and $u_{3}\leq u_{4}$, and all θ,

$$f(u_{1}-u_{3},\theta )f(u_{2}-u_{4},\theta )\geq f(u_{1}-u_{4},\theta )f(u_{2}-u_{3},\theta ).$$

Let the convolution f∗g be defined as

(f∗g)(u,θ)=∫f(u−v,θ)g(v,θ)dv.

Here, the importance of the convolution stems from the fact that the density (or mass function) of the sum U+V of two independent random variables U and V is described by the convolution of their densities.

Theorem 1 *The convolution* f∗g *is both TP and PF, if each of the functions* f *and* g *are both TP and PF*.

(Ghurye & Wallace, 1959)

To appreciate the scope of Theorem 1, consider U and V to be the real‐valued random variables with densities f(u,θ) and g(v,θ), that are independent, conditional on Θ. That is, for all values u and v, and all θ,

P[U≤u,V≤v|Θ=θ]=P[U≤u|Θ=θ]P[V≤v|Θ=θ].

If each of the functions f and g are both TP and PF, then according to Theorem 1, so is the density (f∗g)(u+v,θ). Next, consider the random variables $X_{1},\text{…},X_{n}$ that are independent, conditional on Θ, and have densities that are both TP and PF. Then, sequentially taking $U=X_{1}+\text{⋯}+X_{i-1}$ and $V=X_{i}$, for i=2,…,n, yields that f(s,θ) is TP (and PF), for $S=X_{1}+\text{…}+X_{n}$. Finally, if f(s,θ) is TP, then this is said to correspond to an MLR of Θ by S.

The reason many of the polytomous latent variable models do not imply an MLR is that they do not satisfy the PF requirement (Ligtvoet, 2012). The *rating scale model* (Andrich, 1978) is an example of a model that satisfies both TP and PF, and therefor implies an MLR. However, Masters' (1982) partial credit model does not imply the PF property, yet it implies an MLR, as it has the sum score as a sufficient statistic for the latent variable. This shows that the conditions in Theorem 1 are not necessary for an MLR, albeit sufficient.

### 2.2 Associated random variables

The weak SOL property proposed by Van der Ark and Bergsma (2010) is less restrictive than an MLR and corresponds to the property of two random variables being *positive quadrant dependent* (PQD; Lehmann, 1966). The two random variables U and V are PDQ, if for all values u and v

(1) P[U≤u,V≤v]≥P[U≤u]P[V≤v],

from which the weak SOL property is obtained by taking U=Θ and $V=X_{1}+\text{…}+X_{n}$.

The second result describes a stochastic ordering in accordance with PQD of any two non‐decreasing functions g and h. Here, the function g pertains to the *associated* (multi‐dimensional) random vector $\Theta =(\Theta_{1},\text{…},\Theta_{d})$. The function $g:{\mathbb{R}}^{d}\to \mathbb{R}$ is said to be non‐decreasing, whenever g(θ) is non‐decreasing in each element $\theta_{1},\text{…},\theta_{d}$, for all θ. The function h(X) defined on $X=(X_{1},\text{…},X_{n})$ can be interpreted as any test score that is non‐decreasing in the item scores, which includes but is not restricted to the sum score. For example, h(X) can denote any discretization of the sum score or the positively weighted average $(a_{1}X_{1}+\text{⋯}+a_{n}X_{n})/n$, for fixed $a_{i}\geq 0$ (Ligtvoet, 2022a; Rosenbaum, 1984).

First, to describe the dependencies between the random item variables, we say that $X_{1},\text{…},X_{n}$ are *conditionally independent* (CI), given Θ, if

$$P[X_{1}\leq x_{1},\text{…},X_{n}\leq x_{n}|\Theta =\theta ]=P[X_{1}\leq x_{1}|\Theta =\theta ]\text{⋯}P[X_{n}\leq x_{n}|\Theta =\theta ],$$

for all $x_{1},\text{…},x_{n}$ and θ. Also, we say that the assumption of *monotonicity* (M) is satisfied, if

$$P[X_{i}>x_{i}|\Theta =\theta ]\;\text{are non‐decreasing in}\;\theta ,$$

for all $x_{i}$ and i=1,…,n. Assumption M relaxes the TP requirement for an MLR (Holland & Rosenbaum, 1986). Second, we say that the random vector U is associated (Esary et al., 1967), if

Cov[g(U),h(U)]≥0,

for any non‐decreasing functions g,h. The following result was proven by Jogdeo (1978).

Theorem 2 *The random vector* U=(X,Θ) *is associated, whenever the following three conditions hold*:(Jogdeo, 1978)

a. Θ *is associated,*
b. X *is conditionally associated given* Θ, *and*
c. E[h(X)|Θ=θ] *is non‐decreasing in* θ, *for any non‐decreasing function* h.

The condition (b) is implied by CI (Esary et al., 1967, theorem 2.1), and CI and M together imply condition (c) (Holland & Rosenbaum, 1986, lemma 2). Hence, CI and M, together with (a) imply that (X,Θ) is associated. Also, as any subset of associated random variables is associated, CI, M, and Θ being associated, imply that X is also associated (Holland & Rosenbaum, 1986, theorem 8). Finally, (X,Θ) associated implies that Cov[g(Θ),h(X)]≥0, for all g,h non‐decreasing, which in turn implies PQD, as can be obtain from Esary et al. (1967, theorem 4.4), by taking U=g(Θ) and V=h(X).

The above shows that, for any latent variable model for which CI and M are satisfied, and Θ is associated, PQD holds for any U=g(Θ) and V=h(X) in (1), with g,h non‐decreasing. This mean that for any latent variable model for which these conditions are satisfied, any test score h(X) that is a non‐decreasing function of the item variables (including the sum score) provides a weak stochastic ordering of any non‐decreasing function of the latent variables.

## 3 DISCUSSION

Theorem 2 generalizes the condition that allow for stochastic inferences to be made about latent variables to any non‐decreasing function of the observable random variables $X_{1},\text{…},X_{n}$. These conditions for a PQD are not confined to unidimensional latent variable models, but apply to multiple latent variables that are associated. Examples of such models include the multiple factor analysis model and hierarchical factor model, with non‐negative factor loadings and non‐negative correlations between the latent factor (Ellis, 2015; Krijnen, 2004). The assumption that the latent variables $\Theta_{1},\text{…},\Theta_{d}$ are associated, together with M and CI, means that any non‐decreasing function of these latent variables is positively related to any test score h(X) in terms of a PQD. In turn, the same test score allows for stochastic inferences in terms of a PQD about any one of the latent variables $\Theta_{1},\text{…},\Theta_{d}$. So, under the conditions in Theorem 2, the test score does not reveal anything about the latent structure other than that any non‐decreasing function of these latent variables has a covariance with the test score that is non‐negative; a minimal requirement of a test score to be considered useful. Additional (parametric) assumptions would therefore be required to make specific statements about the structure of the latent variables that account for the dependencies between the item score variables (e.g., Ellis et al., 2025; Ellis & Sijtsma, 2023). But whichever additional restrictions are imposed on the latent structure, these should agree with the conditions in Theorem 2 in order for the test score to be considered useful for making inferences about the ordering on the latent variables.

Although PQD is a weaker version of SOL, the generality of the conditions under which it allows for inferences of the latent variables from the observed item scores places the sum score, or indeed any test score, well beyond a mere intuitive measure or a relic from classical test theory. In their discussion on the use of the sum score, McNeish and Wolf (2020) mention that their goal is *“to raise awareness that sum scoring requires rather strict constraints, imposing these constraints requires the same type of justification as any other latent variable model, and sum scoring corresponds to a statistical model and is not a model‐free arithmetic calculation”* (p. 2287). The results of Theorem 2 largely agree with this assessment, but show that the conditions that allow for the use of the sum score for making inferences are far more general that those considered in their paper. The conditions for a PQD also imply the observable property that X is associated. A test for the item scores being associated could provide an empirical justification for the use of the sum score. Unfortunately, this property is hard to directly ascertain in practice (Ligtvoet, 2022b; Walkup, 1968) and in need of further investigation (Ligtvoet, 2023).

## AUTHOR CONTRIBUTIONS

**Rudy Ligtvoet:** writing – original draft; writing – review and editing; validation; methodology; conceptualization; investigation; formal analysis.

## CONFLICT OF INTEREST STATEMENT

There are no conflicts of interest.

## DISCLOSURE OF ARTIFICIAL INTELLIGENCE‐GENERATED CONTENT (AIGC) TOOLS

No AIGC tools were used.

## Data Availability

Data sharing not applicable to this article as no datasets were generated or analysed during the current study.
